# Acculturation in context: knowledge sharing through ubiquitous technologies

**DOI:** 10.1186/s41039-015-0017-x

**Published:** 2015-10-21

**Authors:** Steven A. Cook, Hiroaki Ogata, Mark G. Elwell, Mitsuru Ikeda

**Affiliations:** 1 Japan Advanced Institute of Science and Technology, Ishikawa, Japan; 2 Kyushu University, Kyushu, Japan; 3 Hakusan International School, Ishikawa, Japan

**Keywords:** Acculturation, Ubiquitous computing, Mobile technology

## Abstract

In this paper, we assess the feasibility of using a retooled mobile and ubiquitous computer system to facilitate knowledge dissemination between users during the process of acculturation. Focused on the foreign population of a Japanese post-graduate university, the system provides a platform on which to study not only the behavior of participants but also the process of acculturation dynamically in context. Both quantitative and qualitative data were analyzed, with quantitative data being obtained through the use of survey instruments based on proven instruments in the field of acculturation and qualitative data obtained via the use of semi-structured interviews. The results of this study demonstrate that the retooled mobile and ubiquitous computer system used had measurable benefit in aiding participants undergoing the process of acculturation.

## Background

The background section of this paper will be divided into two main sub-sections. As this study assesses post-graduate students in relation to the acculturation process, and also the feasibility of using a retooled mobile and ubiquitous system that supports this process, the background section will be divided accordingly.

### Acculturation

The term ‘acculturation’ explains the process of cultural and psychological change that results in the meeting between cultures (Sam and Berry 2010). These changes, typically within a dedicated or fixed context/environment, have a considerable impact on not only the individuals trying to adapt to the culture but also the culture itself. Often looked at as a bottom-up process, in which interactions between individuals experiencing the acculturation process have an impact on what becomes social norms in the future (Erez and Gati 2004), this process varies between individuals. These variations are not only attributed to personality traits or characteristics, but in addition, the experiences individuals are subjected to over time while adapting to a particular context and/or environment. Acculturation is seen as powerful in relation to the psychological and behavioral outcomes and attitudes of individuals going through this process (Smart and Smart 1995; Sam and Berry 2010), and understanding acculturation levels is seen to be beneficial in the application of important organizational, clinical, and social services (Nagata 1994; Puente and Garcia 2000; Thom-Santelli et al. 2011).

#### Psychological and cultural change

At the cultural level, changes occur in relation to social interactions, collective activities, and systems of institutional structure. As an individual adjusts to a new cultural context, regardless of if the context is organizational or purely cultural from the traditional sense, the individual adapts to these new settings, thus forming change in relation to how he/she interacts. These changes occur in several different ways, for example, the way in which individuals acquire language and communication skills, attitude adjustment towards interaction strategy in certain social situations, changes in relation to food preference, and also changes in relation to how one wears his/her hair or even how he/she may dress. It has also been said that group change may also occur amongst those acculturating (Berry et al. 1986; Peñaloza 1994; Matsudaira 2006), and that these new group dynamics, albeit difficult to measure and often overlooked, have an impact amongst all participants within the context/environment (Berry 2006; Thomson and Hoffman-Goetz 2009).

At a psychological level, an impact occurs in relation to the change of behavioral characteristics of individuals acculturating. As individuals are increasingly more immersed in the dominant or host culture, their experiences within this context have a deeper psychological impact (Lefley 1976; Berry 1980, 1989; Erez and Gati 2004; Gim Chung et al. 2004). Not only do individuals question their own personal identity via reflection of their own activities throughout any given day but they also formulate questions relating to their own culture and the possible relationship between the dominant or host culture in which they are immersed. This process is said to be the catalyst behind many stresses and issues not only within the individual but also in relation to contextual changes associated with the host culture. These stresses are said to have an impact in relation to productivity and day-to-day function within the new cultural context or environment (Sun 2013; Stevens and Lee 2002; Stevens and Vollebergh 2008; Sam and Eide 1991; Padilla et al. 1985). Again, from the psychological perspective, other research indicates the need to focus on the environment in which persons acculturating are placed (Stevens and Vollebergh 2008). Contextual and cultural differences surrounding policy, the ethnic breakdown of the local environment, and the perceived ease of returning to one’s own country of origin all have an impact (Hull 1979).

#### Stresses and impact

Psychological health being affected in a detrimental manner has been demonstrated to be a frequent occurrence for individuals during the process of acculturation in a foreign country (Zheng and Berry 1991; Sam and Eide 1991; Stevens and Lee 2002; Padilla et al. 1985; Ben-Sira 1997; Douglas and Roberts 2000; Tilburg and Vingerhoets 2005; Mirdal 2006; Gertzog 2011; Valencia-Garcia et al. 2012; Benet-Martinez 2006; Hull 1979). Expectations about a country, the people, and culture within its borders and their perceived status or position also have an impact, and this is even before individuals reach the shores of the host or dominant culture (Thomas and Althen 1989; Murphy-Shigematsu 1998). These expectations may be based on rudimentary stereotypes generated from media in the individuals’ own home country or based on the individuals’ upbringing or friends’ opinions.

These stresses are often surrounding language acquisition, cultural communication, and feelings of isolation (Padilla et al. 1985; Ben-Sira 1997; Douglas and Roberts 2000; Tilburg and Vingerhoets 2005; Mirdal 2006; Gertzog 2011; Valencia-Garcia et al. 2012). Impact in regard to self-esteem has also been discussed (Lefley 1976). Research indicates that these issues are contextual in nature and are also based on the experiences of the individual and/or groups within the context itself (Portes and Rumbaut 2006). Contextual relevance is important in knowledge acquisition and sharing (Volet 1999, Hwang et al. 2008; Gertler 2003), and thus it stands to reason that it is important in relation to the acculturation process.

Issues surrounding the stress associated with the process of acculturation have been said to lead to low productivity (Louis 1990; Kompler 1999) and even higher mortality rates amongst the foreign population within its host country (Stevens and Lee 2002). The study by Stevens and Lee (2002) makes assertions to a possible correlation between the difficulties associated with acculturating to a new environment, with infant mortality rates amongst foreign mothers within a dominant host culture. These assertions are based on a number of factors: (1) the absence of a concrete regulation in regard to foreign mothers, (2) the absence of informal support groups, and (3) issues associated with acculturation as a whole which covers language, identity, and ethnicity.

#### The four facets of acculturation at an individual level

The process of acculturation is commonly divided into four distinct areas by which individuals undergoing this process are categorized (Berry 1980; Sam and Berry 2010; Sam and Eide 1991; Tilburg and Vingerhoets 2005). These categories provide insight into acculturation balance, thus allowing scholars, psychologists, administrative personnel, and governmental bodies to assess how individuals may be adapting to the new cultural context or environment in which they are placed. These four categories are commonly divided as follows.

From the four categories listed in Table 1, ‘integration’ is seen as the most favorable method by which to acculturate. Integration is seen as the path by which true biculturalism occurs (Sam and Berry 2010; Barry 2001; Berry 1980).

**Table 1:** The four facets of acculturation at the individual level

Assimilation	Assimilation at the individual level occurs when the individual abandons his/her own culture in favor of the host culture and society
Separation	When individuals reject or abandon the host culture and society in favor of preserving their original culture
Integration	Integration occurs when individuals are able to adopt the cultural norms of the host culture while maintaining their culture of origin
Marginalization	Marginalization occurs when individuals reject both their culture of origin and the dominant host culture

Scholars that are focusing on the process of acculturation often have similarity in the instruments used to assess the acculturation balance of individuals. The most common way to assess these levels is by a series of well-constructed questions and statements that require an individual to state agreement/disagreement to the statements provided. Taras (2012), has constructed a catalog of various instruments and tools used for measuring acculturation. This catalog provides insight into various instruments looking at evaluating language, identity, and four-facet acculturation balance. These instruments have been used with success within the field (Benet-Martinez 2006; Gim Chung et al. 2004; Lim et al. 2002) and empirical results verify that these instruments are being used with an acceptable degree of error (Barry 2001).

#### Context, reflective practice, and metacognition

Contextual relevance relating to experiences is seen as important for not only effective knowledge transfer and internalization but also the way in which these individuals share this knowledge in a particular social construct (Gertler 2003; Glisby and Holden 2003). In addition, acculturation often occurs from a bottom-up perspective (Erez and Gati 2004), in which experiences occur in context with peers.

Reflection and metacognition related to the experiences one may have during the acculturation process are other areas that are seen as relevant to the success of these individuals ‘integrating’ to this new context (Helms-Lorenz and Jacobse 2008). As one reflects on a certain experience within the host culture, he/she tends to review possible ways in which the experience may have been guided based on his/her own actions (Exposito and Favela 2003). In addition, when combined with metacognition, individuals also look towards their own thought processes in relation to this experience, plan a strategy, monitor and evaluate this plan, then repeat this process to obtain a level of knowledge adequate in this context.

### Mobile and ubiquitous technology

Mobile and ubiquitous technologies differ in that mobile computing is defined as computing that can be taken with you and ubiquitous technology/computing is computing in varying iterations that appears anywhere and everywhere in a seamless and transparent way (Rodríguez and Favela 2003). Both have become hot topics in the recent times (Blanchard and Mizoguchi 2014; Ling 2004; Stockwell 2007; Sharples 2000; Srivastava 2004; Wentzel 2005) and also are seen as becoming increasingly more important (Sousa et al. 2006).

The term “ubiquitous computing”, put forward by Xerox Palo Alto Research Center employee Mark Weiser, is often seen as technology reliant on an interdisciplinary and complex network of information and technology (Mühlhäuser 2008). This technology relates to not only robust and affordable sensors that are embedded into a particular context or environment but also the computer terminals themselves used to interface with these sensors.

In many ways, ubiquitous technology differs from regular forms of computer interaction, in that it may not need a traditional GUI (graphical user interface) for users to interact with the system. An example of this that we are all used to would be RFID (radio-frequency identification) chips embedded into driver’s licenses, passports, or ID cards. As users slide their cards over a terminal, certain information can be either retrieved from or shared with the computer system itself, thus leading to a more automated and user-friendly experience. Strategy around the creation of ubiquitous computing frameworks is often aimed at embedding technology around us in a very organic and “human” way (Abowd and Mynatt 2000).

#### Benefit of ubiquitous computing in terms of reflection and metacognition 

As mentioned previously, reflection and metacognition are seen as important within individuals undergoing the acculturation process (Helms-Lorenz and Jacobse 2008). It has also been demonstrated as important in educational settings (McCrindle and Christensen 1995; Suwan and White 1994; Daudelin 1996). Ogata et al. (2010, 2004) demonstrate that combining a journal writing method aimed to promote reflection and metacognition, with mobile and ubiquitous computing, users obtain benefit not available previously. Not only does this technology provide access to previously recorded externalizations of users, it also provides automatic contextual relevance via GPS, RFID, and quick response (QR) codes. By using these technologies not only to externalize the experiences one has within the context itself (via video, sound, and photos) but also have these experiences shared with others within the same context, it is demonstrated that these users are able to reflect in a new and powerful way.

The metacognitive and reflective benefit in context is an attribute shared amongst a number of other studies using this technology (Hodges et al. 2006; Hwang et al. 2008; Hou et al. 2012; Ogata et al. 2010; Ogata and Yano 2004). Being able to apply reasoning techniques while being physically located/co-located within a certain context has a benefit in regard to how one reflects upon his/her actions, plans a strategy for future actions, and understands or empathizes with others in the same context.

## Methods

### Strategy

#### Stage 1—initial survey and interviews

The first stage of our research strategy consisted of a survey given to post-graduate students at the Japan Advanced Institute of Science and Technology (JAIST) to quantitatively assess base acculturation levels (assimilation, integration, marginalization, and separation), language ability, and also the time duration spent in Japan/the post-graduate university. Previous research demonstrates effectiveness for gauging the acculturation balance (Barry 2001; Benet-Martinez 2006; Gim Chung et al. 2004; Lim et al. 2002), and the surveys used for this study are based on these instruments.

This initial survey was given to the 24 participating post-graduate students coming from America, Thailand, Vietnam, China, Taiwan, Mongolia, and Bangladesh. These students ranged in age from 23 to 35 years of age, had varying levels of Japanese/English ability, and had spent between 1 month and 5 years in Japan.

Semi-structured interviews were also given after completion of the first survey to qualitatively look at the issues these students were having within the post-graduate institution/Japan and also to gain insight into the preconceived notions of what participants expected of Japanese life. By cross-referencing the information gathered from the individuals during the interview process with the survey data, the interviewees are also looked at in relation to constancy of answers.

The questions asked were as follows:

- What did you know about the Japanese/JAIST lifestyle before coming to Japan?
- Did your expectations match reality?
- Have you had any issues surrounding cultural difference (either in JAIST or Japan in general)?

#### Stage 2—participant selection for the ubiquitous system

Based on the results of the stage 1 survey, the participants were selected for using the mobile and Ubiquitous Learning Log (ULL) system. The selected participants must have had an acculturation balance outside of what is considered to be the most favorable (integration) and in addition, been already familiar with Android-based technology.

Two functioning groups were then created—‘group A’ and ‘group B’. Group A was not using the ubiquitous technology and acculturated as they would normally. Group B used the ubiquitous system and, as such, acculturated with the assistance of the technology.

In total, there were 19 students acculturating without the use of the ubiquitous system, and 5 acculturating with the aid of the ubiquitous system. The size and balance of the sample was affected by issues of available resources and familiarity with the mobile technology being used.

#### Stage 3—second survey and interviews

After a 3-month period, a second survey was given to all respondents of the first survey—in other words, to both group A (who were acculturating unassisted without using the ubiquitous system) and group B (who were using the system to help them through the acculturation process). This survey, again, was based on proven instruments in the field and was aimed at obtaining acculturation balance change for both groups (group A not using the system and group B using the system).

Semi-structured interviews were also conducted, but these interviews differed from the interviews given after the first survey, in that these interviews were only given to those who had used the mobile and ubiquitous system. In addition, these interviews differed as they were aimed at gathering qualitative data surrounding the use of the ubiquitous system itself, whereas the first round of semi-structured interviews were aimed at understanding acculturation difficulties in more depth.

The questions asked were as follows:

- Has the system helped you to understand yourself and others in any way?
- What kinds of practical issues did you have using the system (if any)?
- Does a system such as this have benefit over other kinds of communication technology in this context?
- Do you feel that you’ve learned something about Japan or JAIST via this system?

#### Stage 4—cross-referencing and analysis

Quantitative data from the first and second surveys was then cross-referenced to search for acculturation balance changes in the participants, thus leading to comparisons surrounding the effectiveness of the system itself. In addition, feedback surrounding the system was looked at in detail.

### The ubiquitous system utilized

This study retooled an existing ubiquitous computer system that is currently used with success in traditional educational frameworks. The base system being used is the “ULL” system developed by Professor Hiroaki Ogata and his team at the Tokushima University (2010). This system facilitates the recording of learning experiences that occur anytime, anyplace. It then allows users to share, reflect upon, and reuse these experiences.

Within the following three sub-sections of this paper, the key functional elements of the existing ULL system that will remain intact for this study are explained.

#### The ULL recorder

The ULL recorder facilitates uploading and storing of learner experiences into the system from wherever or whenever users may find themselves. Using either an Internet browser-based client or an Android application, learners have the ability to take photographs or video and then apply them to certain logs, ask questions in relation to the log, comment or add extra information, apply GPS location information, and allocate a QR code for easy retrieval. In addition to the functions stated above, language translation supporting numerous languages is also built in. In terms of the server side of the system, these logs are stored in a server running on Linux OS, programmed using Java and PostgreSQL.

#### The ULL reminder

**Fig. 1:**
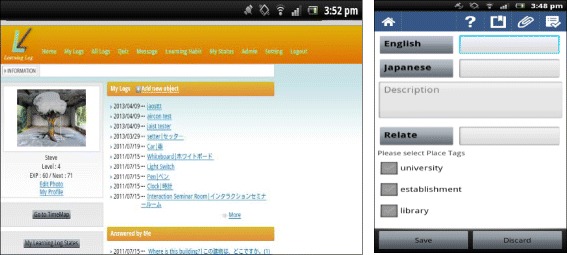
ULL recorder and log review screens

A list of all previous learning logs is stored and organized by the system. Figure 1 highlights the ‘My Log’ section displaying the logs that an individual has uploaded into the system, and this screen can also be used to look at unanswered questions or the externalizations of others. These logs can be automatically displayed when a user enters the same location in which the log was first externalized, or they can be displayed by the user simply looking through logs manually.

#### The ULL navigator

**Fig. 2:**
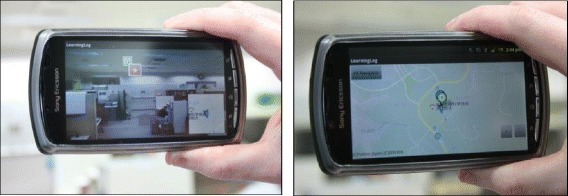
Smartphone augmented reality display and Google Map display

The ULL navigator provides the user with an AR (augmented reality) interface with which to navigate through the ULLs in a real-time contextual manner. When a learner enters a certain geographical location with his/her mobile device, GPS information attached will send an alert through the system to the learner. Once alerted, an AR view will appear clearly highlighting logs that have been recorded in the current area. If a user should decide to click on a certain log, a Google Map will be retrieved marking the ULL object location and in addition, marking the users themselves in relation to the said log. This function allows for context-specific information to be easily shared amongst users of the system. (Fig. 2)

### How this system was used in this study

To assist the reader in understanding how participants are using the system, we put forward one actual externalization from one of the users and in addition, the feedback and responses from other users on the topic. Please keep in mind that the participants were not guided in regard to what kinds of externalizations they should input into the system or the kinds of responses they should provide to questions/comments.

#### Experiences shared from within the environmental context

First of all, a user of the ULL system enters a location within the university context. This could be a particular room or location within the university itself or a location outside of the university walls. The user then has an issue or problem in this context or simply would like to share an experience or ask a question to other users about this context.

**Fig. 3:**
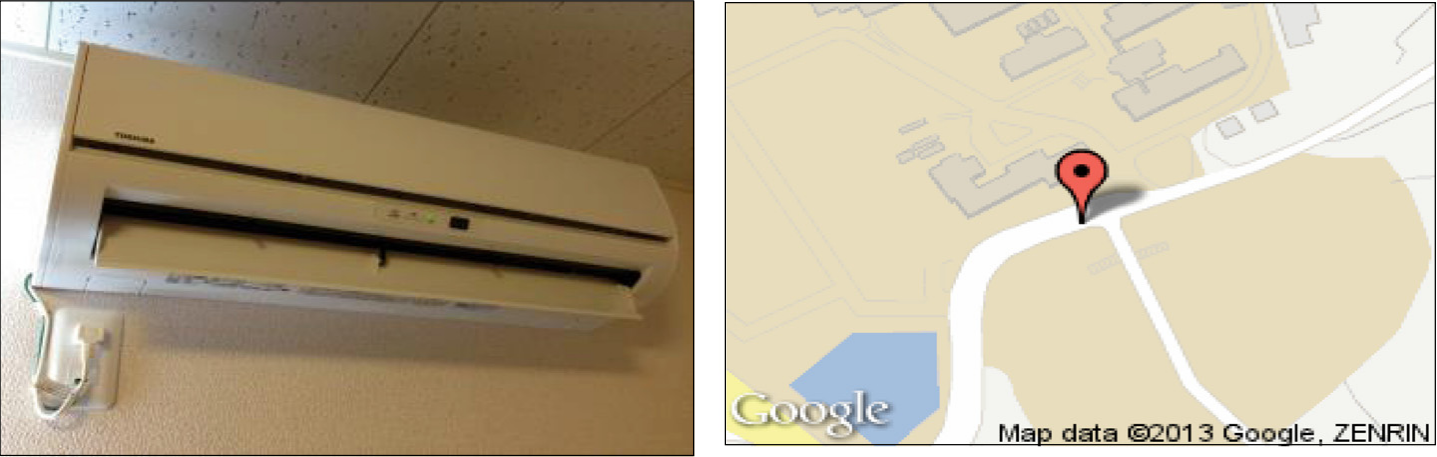
User photograph (*left*). Google Map location (*right*)

In the case we put forward here, a user had a difficulty in relation to the policy surrounding heating systems being switched on in a certain location. In Japan, there is usually a set date for these systems to be activated globally, regardless of the actual outside air temperature, but this user was unaware of this. From within the university, the user then externalized his own opinion on the matter and asked the question “Do you know when they turn on the heater? Such a strange policy!!!” In addition, the user took a photo of the location, and the system automatically logged the GPS location of the user (as seen below in Fig. 3).

#### User reflection and discussion

Now that the experience of the user has been externalized via the ULL system, this experience is now able to be viewed by other users of the system. When other users entered the same geographical location, the GPS tag would trigger their mobile devices into displaying the experience externalized by the first user.

In response to the “air-conditioning policy” externalization, other users commented on how they also had difficulty understanding the policy surrounding this matter too. This leads to one user stating “I asked a Japanese friend about this yesterday, and he said that it is important in Japan to display uniformity and togetherness—this is why school uniforms change, and AC is switched on, regardless.” As the reader can see, this particular topic led to a discussion regarding cultural difference and policy. It is important to note that all of this communication was stored in the system and able to be shared with others using the system from within the context itself.

## Results and discussion

### Survey 1 analysis—acculturation balance and interviews (base acculturation levels before group division)

#### Survey objective and overview

Survey 1, as mentioned in previous sections, was designed to assess base acculturation balance levels (assimilation, marginalization, integration, and separation) of all participating post-graduate students, thus providing a point of reference in relation to all students before implementation of the ubiquitous system. It also provided self-assessed details in relation to student language ability and time spent in Japan.

Survey 1 had a total of 24 respondents. These respondents ranged in age from 22 to 33 years old and were from seven varying countries of origin. All respondents had been in Japan for varying lengths of time—ranging from as little as 1 month to more than 5 years. In addition, the amount of time spent in the post-graduate institution also varied from 1 month to 2 years.

A plurality of the respondents, with a total of 45.8 %, were between the ages of 26–30 years old. Chinese students made up 37.5 % of the total number of the students looked at in the first survey, Vietnam 25 %, Thailand 20.8 %, and the remainder of the nationalities at 4.1 %, respectively.

#### Acculturation balance of survey one respondents

**Fig. 4:**
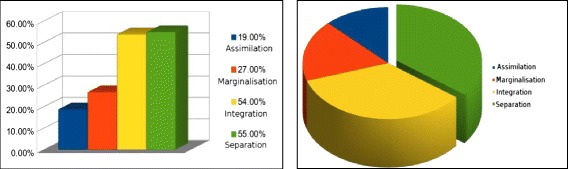
Total acculturation balance of respondents

Figure 4 below shows the acculturation balance of the participants, by presenting the overall percentage of chosen statements in relation to the four areas of acculturation balance (assimilation, marginalization, integration, and separation).

Based on the data provided by survey 1, these results highlight a tendency for the participants to have stronger feelings of ‘separation’ from the post-graduate institute/Japanese culture. To add further detail in relation to feelings of separation, 66.7 % of the respondents agreed with two out of three statements in relation to feelings associated with ‘separation’, and there were not any respondents without feelings of separation at all.

Statements relating to feelings of ‘integration’ (seen as the more desirable form of acculturation balance) also had a considerably high number of participants in agreement; however, the strong agreement with statements relating to ‘separation’ is not favorable. Out of all of the respondents, there were only three that agreed with all three statements relating to feelings of ‘integration’ (three statements being the maximum), but two out of these three respondents also expressed agreement with the two of the three statements associated with ‘separation’. Statements that were relating to feelings of ‘assimilation’ and ‘marginalization’ were amongst the lowest out of the four categories.

#### Language ability correlation

The participants were asked to provide a self-assessment in relation to their language ability to provide possible insight into the correlation of language ability and acculturation balance. English and Japanese ability were assessed using a scale between 1 and 7, with 1 being the lowest skill level possible and 7 being the highest.

In regard to English ability, 50 % of the respondents assessed their own ability at a possible 5 out of 7 or higher, whereas the respondents in relation to their Japanese ability, 25 % assessed themselves at a possible 5 out of 7 or higher. This indicates a definite overall language ability deficit in relation to Japanese language skill.

Using the Pearson’s correlation coefficient, the data does indicate an overall positive correlation between language skill and the ability to integrate into a new environment. However, the data did not demonstrate significant difference between better Japanese or English skill having impact on feelings of ‘integration’ in this study. This may be due to this particular university context having emphasis on a bilingual environment (Table 2).

**Table 2:** Correlation between language ability and integration

		Jap. ability	Int. level
Japanese ability	Pearson correlation	1	0.15
	Sig. (two-tailed)	0.000	0.000
	*N*	24	24
Integration level	Pearson correlation	0.15	1
	Sig. (two-tailed)	0.000	0.000
	*N*	24	24
English ability	Pearson correlation	1	0.14
	Sig. (two-tailed)	0.000	0.000
	*N*	24	24
Integration level	Pearson correlation	0.14	1
	Sig. (two-tailed)	0.000	0.000
	*N*	24	24

In regard to the feelings associated with the acculturation balance of ‘separation’ and ‘assimilation,’ there were correlated differences in relation to the skill levels of each language. These correlations are more in line with what one might expect, with better Japanese skill more likely to lead to assimilation (Table 3).

**Table 3:** Correlation between language ability and separation/assimilation

		Jap. ability	Sep. level
Japanese ability	Pearson correlation	1	0.05
	Sig. (two-tailed)	0.000	0.000
	*N*	24	24
Separation level	Pearson Correlation	0.05	1
	Sig. (two-tailed)	0.000	0.000
	*N*	24	24
		Jap. ability	Ass. level
Japanese ability	Pearson Correlation	1	0.36
	Sig. (two-tailed)	0.000	0.000
	*N*	24	24
Assimilation level	Pearson Correlation	0.36	1
	Sig. (two-tailed)	0.000	0.000
	*N*	24	24
		Eng. ability	Sep. level
English ability	Pearson Correlation	1	0.19
	Sig. (two-tailed)	0.000	0.000
	*N*	24	24
Separation level	Pearson Correlation	0.19	1
	Sig. (two-tailed)	0.000	0.000
	*N*	24	24
		Eng. ability	Ass. level
English ability	Pearson correlation	1	−0.19
	Sig. (two-tailed)	0.000	0.000
	*N*	24	24
Assimilation level	Pearson correlation	−0.19	1
	Sig. (two-tailed)	0.000	0.000
	*N*	24	24

Although the data does reflect some correlation with language ability and acculturation balance, we assert that future work should also look into the personality traits of the individuals themselves.

#### Time spent within Japan correlation

The data gathered indicates that the time respondents have spent in Japan may play some role in the acculturation balance of these individuals (Table 4).

**Table 4:** Correlation between time in Japan and acculturation balance

		Time	Int. level
Time spent in Japan	Pearson correlation	1	0.21
	Sig. (two-tailed)	0.000	0.000
	*N*	24	24
Integration level	Pearson correlation	0.21	1
	Sig. (two-tailed)	0.000	0.000
	*N*	24	24
		Time	Sep. level
Time spent in Japan	Pearson correlation	1	−0.03
	Sig. (two-tailed)	0.000	0.000
	*N*	24	24
Separation level	Pearson correlation	−0.03	1
	Sig. (two-tailed)	0.000	0.000
	*N*	24	24
		Time	Ass. level
Time spent in Japan	Pearson correlation	1	0.06
	Sig. (two-tailed)	0.000	0.000
	*N*	24	24
Assimilation level	Pearson correlation	0.06	1
	Sig. (two-tailed)	0.000	0.000
	*N*	24	24
		Time	Mar. level
Time spent in Japan	Pearson correlation	1	−0.13
	Sig. (two-tailed)	0.000	0.000
	*N*	24	24
Marginalization level	Pearson correlation	−0.13	1
	Sig. (two-tailed)	0.000	0.000
	*N*	24	24

However, it is important to note that the university context in which these respondents live and work is relatively secluded. This may have impact on these results, as it is possible for these individuals to form their own communities and support systems.

#### Interviews—survey 1 respondents

As mentioned previously in the “Strategy” section, semi-structured interviews were given after completion of the first survey. The goal of these interviews was to assess preconceived notions the participants may have had regarding Japan and also to gain an overall perspective of acculturation difficulties within the post-graduate university/Japan.

##### Questions 1 and 2 responses

The first and second questions looked at preconceived notions of Japan and/or JAIST. Based on the literature in the field (Thomas and Althen 1989; Murphy-Shigematsu 2002), it is said that the understanding of people in relation to these preconceived notions has impact on how the individual may interact and participate at the initial stages of acculturation, thus any contradictions may in fact cause the individual to have a delay in relation to acculturation.

All respondents had one commonality in relation to these particular questions. Everyone interviewed commented on how they believed (before coming to Japan) that Japanese people worked hard and for extremely long hours. They believed this work ethic was well ingrained into the Japanese culture, and to be able to effectively work alongside Japanese people, an equal amount of effort was required of them. When asked if this differed from the reality they were presented with on arrival, all agreed that this was in fact how things are done in Japan, and that the Japanese people do have a considerable work ethic. When asked about the pressure associated, many mentioned that the pressure to keep up with this work ethic did present some difficulties; however, the Japanese people within the university did take the additional pressure on foreigners into consideration and were “easy on them” in many situations.

Another area that was discussed was surrounding the university culture and lifestyle itself. JAIST (the post-graduate institution used in this study) prides itself on having an international administrative system and curriculum, and this is often put forward in its public relations information. Many of the interviewees commented that their expectations did not match reality in this regard. It was said that although many activities were conducted in English, there were many other activities conducted in Japanese that they would have liked to be a part of. These activities included seminars, workshops, and meetings. In addition, not all of the classes offered (especially at Master’s level) had English counterparts, thus creating a feeling of discomfort and separation. Many of the students expressed some kind of disappointment and difficulty in this regard.

Another interesting comment brought up by a few of those interviewed was the expectation of the local culture outside of the JAIST campus. Just so the reader is clear, JAIST is located in a relatively rural area, with very green and lush natural woodlands in its surroundings. It is also some distance away from shopping centers and public transport, with a single shuttle bus taking people without a car to the closest small township. Some interviewees were not expecting that JAIST would be quite so “rural”, and their preconceived notions of the location were more akin to that of Tokyo or Osaka. They assumed that there would be many things to do, places to go, restaurants to eat in, and people outside of the university to meet. To some of the students interviewed, there was an expression of disappointment. Others however, expressed relief, as the surroundings, in their opinions, promote study. As mentioned previously, research indicates that acculturation difficulties are common when individuals have discrepancies between their preconceived notions and the reality of a particular context. The individuals talking about these issues did in fact demonstrate this to be the case.

##### Question 3 responses

The third question looked at possible issues surrounding cultural difference between the foreign students and the JAIST or Japanese culture. The interviewees were asked to be extremely candid in their responses.

The majority of those interviewed said that there were feelings of “separation” between themselves and the Japanese people (both inside and outside of the university). This finding was also highlighted in the quantitative survey results. When asked to expand on the possible ways in which this was an issue, many of those interviewed expressed difficulties with “communication” as the main contributing factor. Although face-to-face communication was seen as the most troublesome, interviewees also expressed difficulty with more formal forms of communication, such as email and written reports. Interestingly, even those with considerable Japanese language proficiency also expressed this as a difficulty. Many of those interviewed expressed that the Japanese “shyness” was a considerable hurdle. Others commented on the fact that the “formality” of the Japanese way of doing things provided a metaphorical wall to contend with when dealing with people. Another interesting comment that was also expressed later as a topic externalized in the ubiquitous system was “Japanese people do not say what they feel”.

Issues surrounding rules and regulations also came up as a contributing factor in relation to cultural difference. Some of those interviewed expressed misunderstanding with what was expected of them in relation to certain administrative procedures and, in addition, discomfort when asking administrative and lab staff exactly what is expected of them. However, this was not a commonly agreed upon area, with some interviewees (primarily those who have been in Japan for a longer period of time) stating that rules and regulations were clear, but they are simply not explicitly stated—meaning that a certain level of experience is needed to understand these “implicit” rules.

Friendship was another area commonly discussed amongst the interviewees. However, it must be said that assertions are difficult to make in regard to this being a cultural issue, as of course there could simply be personality traits at play. However, this was a common theme brought up by those interviewed. Many interviewees stated that becoming “good friends” with Japanese people proved to be a difficult and complicated process. This was a common answer to this particular question, and it must be stated that it tended to transcend survey results in relation to marginalization, separation, integration, and assimilation, meaning that most of the students interviewed had difficulties making friendships with Japanese people no matter their acculturation balance.

#### Overall results summary for survey 1 (entire group before group division)

Survey 1 and of course the semi-structured interviews that followed were designed to obtain an overview of the acculturation balance of all students participating in the study. This survey, given before system implementation, provided base acculturation balance levels of all students, thus creating a point of reference with which to compare the impact of the ubiquitous system itself.

In regard to the quantitative data obtained via the survey instrument, the overall results demonstrate that statements relating to ‘separation’ had the highest level of agreement amongst the respondents, with 66 % of the respondents agreeing with at least two statements associated with separation. It is also interesting to note that no students out of the 24 lacked this feeling at all. The interviews also reflect this sentiment, with the majority of the students interviewed expressing some feelings of separation.

The statements relating to ‘integration’ (seen as the more desirable balance of acculturation) were the second in highest level of agreement with 54 % of the participants agreeing with two statements or more relating to this acculturation balance area, and statements in relation to both ‘assimilation’ and ‘marginalization’ were the lowest in relation to agreement. These results indicate that although there was room for improvement, many of the students taking part in the survey were on the right path.

Correlation between language ability and acculturation balance were virtually non-existent. This differs from the majority of literature surrounding this issue, which states the stresses associated with language as a hurdle during the acculturation process. However, based on the points brought up by the students during the semi-structured interviews conducted after the first survey, in which users talked about “communication” being an issue, one could assert a possible difference between communication and language proficiency. Many students commented on the “communication” difficulties between themselves and the Japanese population, both within the university and surrounding areas. The students with reasonably high Japanese language proficiency were amongst these students. This highlights a possible avenue for further research looking into differences with actual communication techniques (relating to culture), rather than simple language proficiency.

Semi-structured interviews also highlighted the differences between preconceived understanding before coming to Japan and the reality of life here itself. Issues surrounding communication, rules and regulations, and friendship were amongst the topics discussed. The qualitative data obtained from these interviews did reflect the quantitative data obtained via the survey instrument, which tended to highlight problems associated with feelings of separation.

Time correlation, as in the amount of time an individual spends in Japan/post-graduate institution in relation with acculturation balance, did not have a significant impact. However, it must be stated that due to the size of this particular study, it is not possible to form definitive statements in this regard and that this issue is something that will have to be further addressed in future work.

Just so the reader is clear, these results above are looking at the base acculturation levels of all students taking part in the study. As mentioned previously, after completion of the first survey/interviews, these respondents were then split into two groups (group A and group B), with group A acculturating from this point on without any assistance from the ubiquitous system and with group B acculturating with the assistance of the ubiquitous system. The following sub-section looks at the results of the second survey/interviews for these two groups.

### Survey 2 analysis—acculturation balance and interviews after 3 months (group A and group B)

As mentioned previously, all of the respondents from survey 1 (the survey designed to provide base acculturation levels) were divided into two groups, with the first group (group A) acculturating as they would normally without the assistance of the ubiquitous system. The second group (group B) will have had access to the ubiquitous system to aid them during this process. The period of time between surveys was 3 months. To reflect this methodology, this sub-section will be organized as follows:

- Sub-section “Group A (acculturating unassisted)—acculturation balance changes—comparisons between the first and second survey” will look at comparisons between the first and second surveys in relation to acculturation balance of only group A (the group acculturating unassisted).
- Sub-section “Group B (acculturating assisted via the ubiquitous system)—acculturation balance changes—comparisons between the first and second survey” will contain comparisons between the first and second surveys in relation to acculturation balance of only group B (the group acculturating via the assistance of the ubiquitous system).
- Sub-section “Comparisons between quantitative data for both group A and group B” will provide the reader with comparisons between the results of group A and group B.
- Sub-section “Group B (acculturating assisted via the ubiquitous system)—interviews” will present the reader with the qualitative data obtained via interviews with only group B (the group acculturating via the assistance of the ubiquitous system), to assess system benefit from the user-perspective.

#### Group A (acculturating unassisted)—acculturation balance changes—comparisons between the first and second survey

**Fig. 5:**
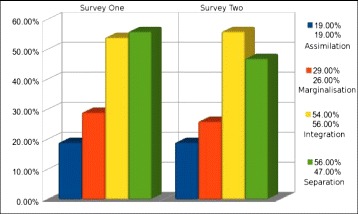
Group A—acculturation balance changes over 3 months

This sub-section presents the comparative results in relation to quantitative acculturation balance data obtained from both survey 1 and survey 2 for only group A (with ‘group A’ acculturating unassisted via the ubiquitous technology). Figure 5 depicts the change that took place over the 3-month period for group A (the group acculturating unassisted), based on the data from survey 1 and survey 2, by comparing the total percentages of chosen statements in regard to the four areas of acculturation balance.

As the reader can see, based on the above Fig. 5, there was change in relation to most of the categories associated with acculturation balance, with reductions of agreement with statements relating to ‘marginalization’ (seeing a 3 % reduction) and ‘separation’ (a 9 % reduction), and also an increase in agreement to ‘integration’ (2 % increase).

To provide more detail for the reader, we will expand this finding by presenting the averages in relation to chosen statements. Please keep in mind that the total number of statements relating to each of the four areas of acculturation balance was three.

Agreement with statements associated with feelings of ‘assimilation’ did not see any change at all; however, ‘separation’ saw a reduction (reduction seen as a favorable direction) from a 1.68 mean/0.48 std. dev. from the first survey to a 1.42 mean/0.51 std. dev. from the second survey. In regard to the feelings/statements in the survey surrounding that of integration (again, the most desirable area of acculturation balance), the participants showed an extremely slight improvement/increase. Integration levels from the first survey for group A had a mean of 1.63/std. dev of 0.83 from the first survey and a 1.68 mean/std. dev. of 0.82. This equates to a single respondent in agreement of one additional statement related to feelings of ‘integration’. The final area of acculturation balance, ‘marginalization,’ saw a slight decrease (decrease being favorable) from a mean of 0.89/std. dev. of 0.66 to a mean of 0.79/std. dev. of 0.71. These findings demonstrate that group A did have desirable change in relation to acculturation balance overall over the 3-month period.

#### Group B (acculturating assisted via the ubiquitous system)—acculturation balance changes—comparisons between first and second survey

**Fig. 6:**
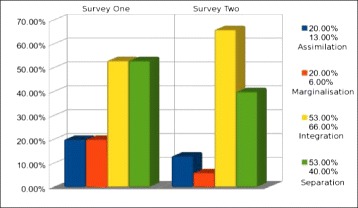
Group B—acculturation balance changes over 3 months

This sub-section looks at the comparative changes of group B (the group that acculturated with the assistance of the ubiquitous system) over the 3-month period. Figure 6 looks at the total percentages of chosen statements of agreement by the participants from both the first and second survey, thus providing overall balance levels.

Figure 6 demonstrates that in regards to total statements agreed with by users of the system, there was a 6 % reduction for ‘assimilation’, 14 % reduction associated with ‘marginalization’, 13 % reduction for ‘separation’, and a 13 % increase with agreement of statements relating to that of ‘integration’.

As with the previous sub-section looking at non-users, we will provide the reader with more details in relation to the averages of chosen statements for the users of the system. Again, the total number of statements relating to each area of acculturation balance is three. Feelings/statements in regard to ‘assimilation’, ‘separation’, and ‘marginalization’ balance were reduced in the participants, and feelings/statements associated with ‘integration’ had an increase (increase seen as favorable). In relation to ‘assimilation’, the original survey yielded a result of the mean being 0.60 with a std. dev. of 0.89, whereas in the post-system survey, these numbers were reduced (with reduction being seen as desirable) to 0.40 and 0.55, respectively. Survey statements in relation to ‘marginalization’ demonstrated a result of the mean being 0.60 with a std. dev. of 0.55, whereas the post-system survey has 0.20 and 0.45, respectively (again, this is a desirable reduction). Statements associated with feelings of ‘separation’ were also reduced with a mean of 1.60 and 1.20, respectively, with a 0.10 variation in regard to the standard deviation. Change in a favorable direction for ‘integration’ was observed, with the original survey yielding a 1.60 mean and a 0.55 standard deviation, and the second survey yielding 2.0 mean with 0.71 standard deviation in the post-system survey.

#### Comparisons between quantitative data for both group A and group B

When comparing the quantitative survey results between group A (acculturating unassisted) and group B (acculturating assisted via the ubiquitous system), these results demonstrate that overall, users of the system saw slightly better improvement in regard to acculturation balance in relation to these surveys.

**Fig. 7:**
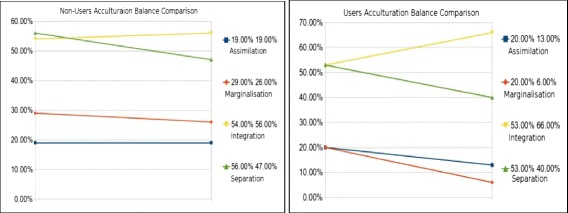
System non-users vs system users acculturation balance comparison

To expand this further, we will present the reader with side-by-side visual representations of the change between the two groups below in Fig. 7. These changes are based on the total percentage of agreed upon statements within the first and second survey instruments and demonstrate the difference in relation to change between the two groups over the 3-month period.

#### Group B (acculturating assisted via the ubiquitous system)—interviews

This sub-section looks at the results gathered from semi-structured interviews given only to users of the ubiquitous system. The goal of these interviews was to gather qualitative data in relation to the effectiveness of the ubiquitous system itself.

##### Question 1 responses

The first question given to the users of the system, ‘Has the system helped you to understand yourself and others in any way?’, was aimed at understanding any possible benefit in relation to the reflective practices of the users when looking at the system. Of course, the question is designed as to not lead answers.

The response from all participants was a definitive “yes” in regard to this question; however, when asked to elaborate, there were variations in regard to how this took place. The users felt that the system gave them a window into the minds of others within the JAIST environment and that being in a position to provide help or simply talk to others about their issues not only filled them with a sense of well-being, it also facilitated a deeper understanding of the issues affecting other foreigners in certain contexts. Three of those interviewed elaborated on this, by making reference to feelings of “togetherness”, “community,” and “common problems” when reading the issues of others that were similar to their own problem in similar situations. This highlights the way in which the users of the system began to bond together to form mutual understanding, thus, one could assert the system as a viable tool to build communities and student self-efficacy.

In relation to the system providing a way to understand themselves, some users expressed the positive way in which the system was able to simply help in sharing their own problems. Externalizing their own problems, while at the same time maintaining a narrow context related focus, provided these students with a way to “get worries out” and also understand where those worries may have come from. The users commented that hearing the feedback from others helped to alleviate concerns they themselves might have had.

Other students expressed a deeper level of reflection occurring via use of the system. When they reviewed their externalizations from within the context itself, they mentioned that they had a better understanding of their own thinking and actions within this context. One student mentioned that reviewing their externalizations on a different day—when they were in a different emotional state—provided a way to see the context in a different light and also to think about their actions and thoughts from a new perspective.

Cultural understanding also was a topic brought up by one of the users of the system. This particular user expressed feelings of “cultural difference” through the experiences of other foreign people within the context. This user felt that he had a better understanding of other foreigners and how they saw certain Japanese cultural attitudes. According to this user, this led to him understanding his “own cultural differences in a deeper way”.

Finally, it was also mentioned as a criticism, that it would be nice to hear from Japanese individuals within the system. This particular study focused on purely the externalizations of foreigners acculturating within the environment, and there were not any Japanese participants. It was said that having Japanese feedback to certain externalizations would be beneficial in understanding the Japanese perspective. This is another area that could be brought up for future work.

##### Question 2 responses

The second question ‘What kinds of practical issues did you have using the system (if any)?’ looks at the system in use. The users were asked to provide any practical issues surrounding the system during the time they were using it. These issues could be related to, but not restricted to, user interface design, bugs, design flaws, or general user friendliness.

In general, the users did not express difficulties that hindered them completely from using the system to externalize their issues, make and comment on questions, or navigate the system. However, there were some issues that did arise with some of the functional aspects of the system. The user interface was one such area. Differences between the browser-based client and the Android-based client did cause confusion for a few of the users. Two users mentioned that they would have liked to see more similarity between these two user interfaces. This was relating more towards the learning curve needed for each client, rather than one being more complicated than the other.

An interesting comment brought up from one user was in relation to the amount of notifications that the system was providing for the mobile/Android application. As mentioned previously, the system provides notifications when users enter certain locations and have questions answered etc., but this particular user wanted “more notifications”. For example, when other users posted a new issue or problem, he wanted the mobile portion of the system to alert all users regardless of their location.

A few users experienced errors when using the system, which did provide a level of frustration in regards to lost comments or externalizations. These errors were mainly in relation to the retooling of the system itself. As not all of the original functionality from the ULL was needed, there were some menu items that were not relevant or fully functional, thus causing some issues. Once the users became accustomed to what was being used and what was not being used, the rate of error reports decreased dramatically. This is something that will be looked into in future studies, in which of course an updated interface design will be implemented.

The final area in which two users expressed difficulty was in relation to the amount of time needed to input externalizations that were detailed and that also contained questions. The original system was designed with vocabulary building in mind, so written paragraphs etc., may have been somewhat difficult to input. Another possible reason for this comes from the fact that not all of the users were familiar with the Android operating system itself.

##### Question 3 responses

This particular question, ‘Does a system such as this have benefit over other kinds of communication technology in this context?’, was created to have users compare other communication mediums, such as Facebook, Twitter, GooglePlus, or BBS (Bulletin Board Services)/forums. We were interested to understand if the users felt differences in relation to this system over more traditional forms of communication technology.

Narrow focus as a benefit was something that was brought up by all users of the system. The system was essentially restricted to the post-graduate institutional context and also a small number of foreign participants, which provided a feeling of “safety” for the students externalizing their issues. The participants mentioned that this was different from that of Facebook or forum-based systems, where friends of friends, or even other users at random, may have access to the externalizations of users. One user commented on this being beneficial in relation to “community building” and “trust” within the system, too. Of course, it must be stated that the number of users for this particular study is on the small side, but it is our assertion that the closed nature of the system in relation to contextual relevance, and also restrictions to outside access, would provide benefit even with user numbers expanded.

Strong contextual relevance was another area brought up by the majority of users. The users understood that the comments given, the issues externalized, and the interactions taking place were all from within the post-graduate university context and that the people behind these utterances were going through similar adjustment difficulties to those they themselves were. In addition, the users felt that “inserting their knowledge” into the area in which the issue occurred (via GPS locator information attached to the logs) was “unique” and “powerful”. Judging from user feedback, being able to read the externalizations of other individuals, from the exact same place in which these externalizations were posted, proved to be quite beneficial in understanding the issues themselves.

##### Question 4 responses

Question 4, ‘Do you feel that you’ve learned something about Japan or JAIST via this system?’, was aimed at looking into what the users may have learned about JAIST or Japan via the system.

Responses to this question varied considerably, and interestingly, most of the users put forward their own externalizations as examples, rather than a general overview of certain topics or areas learned. Having said that, there was feedback from the users in regard to non-specific areas of acquired knowledge via the system. One user, for example, commented on having his “umbrella stolen”, and that based on the feedback from other users, understood that this was not necessarily an act of aggression, but merely a common occurrence in which people tend to treat these items as “public property”—a stolen umbrella may not be considered a crime. Another user commented on an uploaded externalization that talked about Japanese behavior, to which he said “he never thought about it like this before”.

### Additional observations and benefits

#### The knowledge-embedded environment

As reflected in the post-system interviews with the participants, the users found benefit in having their externalizations embedded within the context or environment itself. As mentioned previously, it is said that contextual knowledge acquisition and sharing are seen as important (Hwang et al. 2008, Gertler 2003), and thus, for an individual to effectively acculturate to a new environment, individual’s experiences within a particular context are more effectively shared and reflected upon within that same context. The ULL system uniquely allows knowledge to be shared and disseminated amongst all users of the system but remains attached to the context or environment itself. This is what we call the “knowledge-embedded environment”.

As a user experiences something in the environment that may lead to questions or issues regarding cultural difference, a user simply uploads an externalized form of this experience into the system using the ULL recorder. Along with the uploaded experience/query, GPS location information, printable QR codes, and corresponding augmented reality cues can all be placed in the environment and then subsequently used to retrieve or trigger logs within this same context or location. When the original author, or another user of the system, enters into the environment that has the GPS/QR/RFID information attached with his/her mobile device, their mobile devices will be triggered into displaying the particular issue/comment/question logged by the original author.

**Fig. 8:**
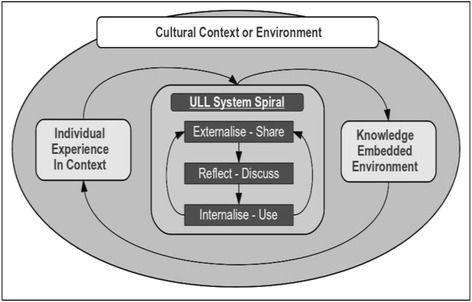
From individual experience to 'knowledge-embedded environment’ (model)

In Fig. 8, it is demonstrated how an individual’s experience is input into the ULL system, then subsequently shared, reflected upon, and internalized. This process remains embedded in the context or environment itself.

#### Dynamic user-generated content

As discussed in the introduction, the process of acculturation is seen as one that is not only extremely dynamic in nature but also occurs from a bottom-up perspective (Erez and Gati 2004). As the users of the system had experiences within the context or environment, these experiences form the knowledge that relates to certain behavioral and cultural changes in the individual. The speed at which changes occur in the environment, or the speed at which changes occur in the individual, highlights difficulties in traditional ways of keeping track of these changes amongst individuals during the acculturation process. Having externalizations happening in real-time and shared in real-time, demonstrated how quickly pertinent information is not only disseminated amongst users, but in addition, how quickly we as researchers could monitor the process. We see this as a considerable benefit over traditional forms of community monitoring methods.

#### Acculturation monitoring in specific geographical locations

**Fig. 9:**
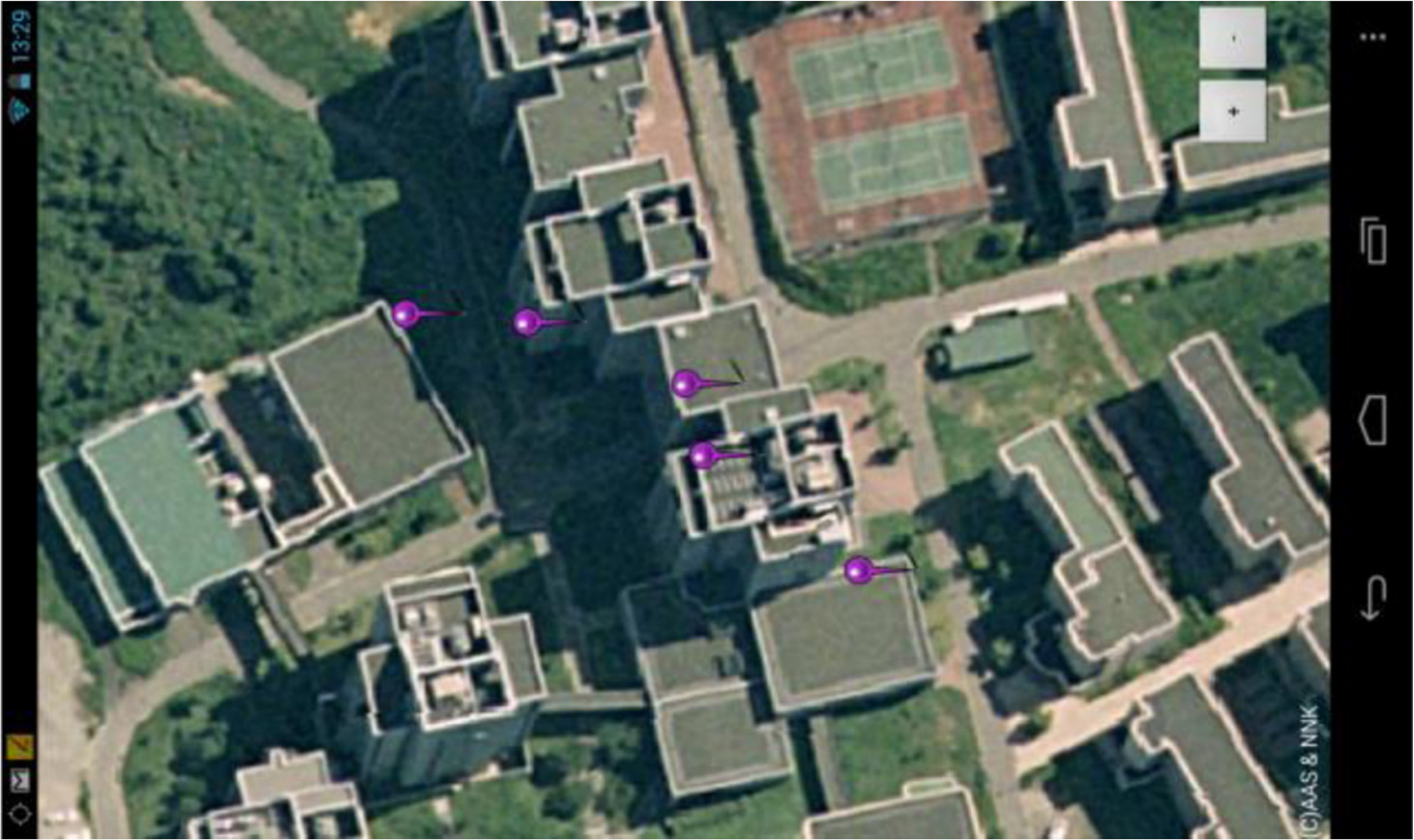
Pinpointing issues within the context itself (Google Maps)

Another benefit of using ubiquitous computing to aid in the process of acculturation comes from the way in which knowledge and behavioral changes can be logged and monitored within the system. Understanding where trouble may have occurred, and looking at the perceptions of individuals surrounding these troubles, is valuable. Figure 9 below is a Google Map image of location points in which some users participating in this study had certain trouble. As is visible in Fig. 9, these points demonstrate how effective this kind of system is for pinpointing issues users may be experiencing in context. By accessing these points, it becomes possible to not only find trouble areas but, in addition, access the issues externalized by the participants within these areas.

#### Deeper knowledge transfer and behavioral trait assessment

As users continued to add to existing log entries and, in addition, made comments or gave advice, the system provided a way to gauge the behavioral change, knowledge level, and progress within these users. Although the time of the study was short, correlation between early logs uploaded into the system and subsequent externalizations provided insight into the reasoning process utilized by the participants in certain situations. To expand on this, when a user had externalized certain experiential and contextual problems into the system and has had comments from others in relation to this problem or issue, this user then commented effectively on the uploaded externalizations of others in similar situations. These interactions were effectively monitored via this system and provided a way to observe these students without invading personal space. Previous research demonstrates the issues associated with observations on student interactions when co-locating the same space (Rodrigo et al. 2014), and thus perhaps, a system such as this may alleviate some of these problems.

## Conclusions

The results of this study demonstrate that the mobile and ubiquitous computer system used had benefit in aiding participants undergoing the process of acculturation. Both the qualitative feedback from user experience and quantitative data associated with acculturation balance have been used to reach this conclusion.

Quantitative data was obtained via the use of two survey instruments. These surveys were based on existing instruments in the field of acculturation and were used to analyze the acculturation balance levels of the individuals taking part in the study. The first survey was given to provide base acculturation balance levels of all participants and also obtain information pertaining to duration of stay and language ability, before implementation of the ubiquitous system. After obtaining base acculturation balance levels, all respondents were then divided into two groups—with one group using the ubiquitous technology, and one group acculturating unassisted. The second survey was given after a 3-month period to both users and non-users of the mobile and ubiquitous system. The second survey provided a method by which to assess and compare change in acculturation balance levels in the participating students.

Qualitative data was obtained via two rounds of semi-structured interviews. The first round of interviews was conducted after the first survey and was designed to gain further insight into the possible acculturation issues the respondents may have been experiencing within the post-graduate university context. The second round of interviews was given only to the users of the ubiquitous system and was designed to gather qualitative data in regards to the effectiveness of the system itself.

When cross-referencing the acculturation balance results from the two survey instruments between the users and non-users of the ubiquitous system, quantitative data demonstrated that the users of the ubiquitous system had stronger levels of agreement with statements relating to ‘integration’ (seen as the most favorable acculturation balance). In addition, the users of the system saw a decrease in ‘assimilation,’ ‘separation,’ and “marginalization” over their non-user counterparts. However, due to the size and duration of the study, it is difficult to make the assertion that the system played a role in this change. This change may have merely been a fluctuation in data.

Qualitative data obtained via semi-structured interviews not only demonstrated that users of the ubiquitous system saw benefit in using the technology for this purpose, it also highlighted the fact that the system may alleviate some of the issues put forward from the first round of interviews, thus having benefit for this purpose. Some common themes brought up by those using the system in the second round of interviews were:

1. The way in which the system facilitated their own reflective practices
2. How the system provided narrow focused discussion surrounding particular issues
3. The way in which the system facilitated strong contextual relevance

The users of the system talked about how they were able to use the system to better understand themselves and others during this process, share with others from within the narrow university context, and finally, build a community with similar issues.

There were some issues in regard to the study however. The number of participants and time duration both need to be increased to alleviate possible deficiencies in data analysis methods. In regard to the ubiquitous system itself, some practical difficulties were expressed by users. Issues relating to differences between the user interface of the Android-based application and the browser-based application were discussed. Being that the system uses a slightly different user interface for both the Android-based application and the browser-based application, some users expressed an overly steep learning curve when first coming to grips with how the system works in practice. Other issues brought up in the interviews were surrounding some of the functional elements of the system itself. For example, the amount of time needed to input certain information in the relevant fields allocated was somewhat lengthy. We assert that this is mainly due to the fact that the original use for the system was primarily designed for vocabulary building and not the externalization of acculturation difficulties. In addition, the occasional system glitch at the beginning of the study created a few problems for users, again, due to the new way in which the system was used for this study.

### Future work

There are a number of areas in which this study should be further expanded upon. First and foremost, it would be beneficial to increase the number of participants using the ubiquitous computing system. One user of the ubiquitous system, during the semi-structured interview, stated “although understanding people from other cultures was achieved via the system, having more people able to comment or provide different perspectives would have been nice”. In addition, an increase in participants would provide better understanding of common or reoccurring issues within particular cultures, more stability surrounding the correlation between language ability and time duration, and also more data with which to apply repeated measures ANOVA calculations or *t* tests to more accurately measure change beyond simple statistical analysis.

A second area in which future work should be conducted is in regard to the personality trait and character assessment of the individuals going through the process of acculturation. Having a better understanding of the personality traits of the individuals themselves (in relation to susceptibility of stress-related issues) would shed light on how these participants progress during their time in the university. This would also provide another mechanism with which to search for possible correlation between the time spent in context and the rate at which acculturation progression occurs.

In addition, an increase of study duration would benefit greatly towards the understanding of acculturation in general. Although the 3-month period of this study did yield results, following along with students at regular intervals over a longer time period would also facilitate the monitoring of specific troubled individuals and even perhaps shed light as to why these difficulties are occurring.

Based on interviews with the users of the system, a redesigning of the interface may also benefit future work. Although interface difficulties did not hinder users in externalizing their issues, it was made clear by some that the differences between the Android-based application and the browser-based application provided complications. Again, it must be reiterated that the system used for this study was based on an existing system originally used for a completely different purpose, and thus it stands to reason that a redesign of the user interface would be of benefit in future studies surrounding the process of acculturation.

Another possible way in which a redesigning of the ubiquitous system could benefit future work would be related to the ‘context’ itself. At present, the system looks at only the geographical context of the users, but as we know, these geographical locations may vary depending on the time of day or if the locations are being used for formal or informal activities. Redesigning the system to take this into consideration would give insight into different layers of ‘context’ within a geographical location.
